# Rate of manual leukocyte differentials in dog, cat and horse blood samples using ADVIA 120 cytograms

**DOI:** 10.1186/1746-6148-10-125

**Published:** 2014-06-05

**Authors:** Martina Stirn, Andreas Moritz, Natali Bauer

**Affiliations:** 1 Department of Veterinary Clinical Sciences, Clinical Pathology and Clinical Pathophysiology, Justus-Liebig University Giessen, Frankfurterstr. 126, Giessen 35392, Germany; 2 Clinical laboratory, University of Zurich, Vetsuisse Faculty, University Zurich, Winterthurerstr. 260, CH-8057 Zurich, Switzerland

**Keywords:** White blood cells, Cytograms, Blood smear review, Laboratory productivity, Left shift, Reactive lymphocytes, Dog, Cat, Horse

## Abstract

**Background:**

Modern automated haematology instruments are capable of performing leukocyte differentials faster, cheaper and with a higher precision than the traditional 100-cell manual differential count. Thus, in human laboratories, criteria are defined for performing a manual review of the blood smear resulting in a marked reduction of manual differential counts. While common in human laboratories, this approach to reducing the number of manual differentials in veterinary laboratories is still not commonly performed. Thus, our aim was to determine the rate and causes of manual leukocyte differentials in a university clinical pathology laboratory using the automated laser-based haematology analyser ADVIA 120. Overall, 14,953 complete blood cell counts from dogs, cats and horses were reviewed. Manual leukocyte differentials were requested if abnormal ADVIA peroxidase and baso cytograms were detected (i.e. suspicion of left shift or atypical lymphocytes/blasts, inappropriate separation of cell populations).

**Results:**

In 21% of canine, 32% of feline and 20% of equine samples, a manual differential was requested. Indistinct separation of the cell population was present in 10% to 15% of the cases. Depending on the species, atypical lymphocytes were suspected in 2% to 12%, left shift in 13% to 25% and suspicion of blasts was present in less than 0.4% of the cases.

**Conclusions:**

The obtained results are comparable to those published for human medicine and the rate of manual differentiation could be markedly reduced in veterinary laboratories if microscopic examination was used as a validation procedure rather than as a reflexive substitute for automated differentiation.

## Background

Several modern large automated haematology instruments [1-8] as well as in-house laser-based systems [9,10] that were originally developed for human medicine have been adapted to veterinary species in recent years. These systems analyse thousands of leukocytes and hence are capable of performing leukocyte differentials faster, cheaper and with a higher precision than the traditional 100-cell manual differential count. Performing a manual review of the slide when the instrument yields identical results reduces the efficiency and productivity of a laboratory and increases the imprecision of the results [11,12]. While the approach to reducing the number of manual differentials is already common in human laboratories, manual differentials are still commonly performed in veterinary laboratories irrespective of the automated count. Veterinary experts’ opinions concerning manual blood film reviews and manual leukocyte differentials are diverse. Whereas, in some places, manual leukocyte differentials are performed for every single sample, other laboratories scan every blood film and only perform manual leukocyte differentials if abnormal cell populations are present or if the automated leukocyte differential appears inaccurate. Besides these approaches, cytograms, which are routinely provided by haematology analysers, can be used to screen for abnormal cell populations and morphology [13] and to decide whether manual review and differentiation is necessary. Reviewing the cytograms seems to be especially important in veterinary medicine as the flag algorithms are not as well established or as comprehensive as those used in human medicine [1]. Early studies evaluating the Technicon H-1E haematology analyser described cytogram abnormalities associated with specific morphological white blood cell changes [14,15]. In human patients, criteria for action following automated complete blood cell counts (CBCs) and white blood cell (WBC) differential analysis are more standardised and advanced than in veterinary medicine [16,17]. A large human multicentre study reviewing more than 90,000 CBCs from 263 institutions showed that manual reviews were performed in 16.2% of cases, including 6.5% manual blood film scans and 9.7% manual differential counts [11]. It was demonstrated that patient care and laboratory operations can be optimised by using manual microscopic examination as a validation procedure rather than as a reflexive substitute for automated methods [13]. The authors also stated that there is no clinical rationale for reflex performance of manual leukocyte differentials based solely on instrument warnings [13]. To our knowledge, there are currently no comparable studies in veterinary medicine. Therefore, the aim of our study was to determine the rates of and reasons for manual leukocyte differentials in our laboratory based on defined criteria for request of manual leukocyte differentials.

## Methods

Overall, 14,953 consecutive blood samples (dog, cat and horse) submitted to the Central Laboratory of the Department of Veterinary Clinical Sciences, Justus-Liebig University Giessen, Germany, for CBC between August 2004 and December 2006 were included. Specimens were received from the Small Animal Clinic, Internal Medicine and Surgery, Justus-Liebig University Giessen, Germany and the Clinic for Horses, Internal Medicine and Surgery, Justus-Liebig University Giessen, Germany.

A specific ethical approval was not necessary as the study was performed retrospectively on samples of diseased dogs, cats and horses routinely submitted to the veterinary diagnostic laboratory for diagnostic workup.

**Figure 1:**
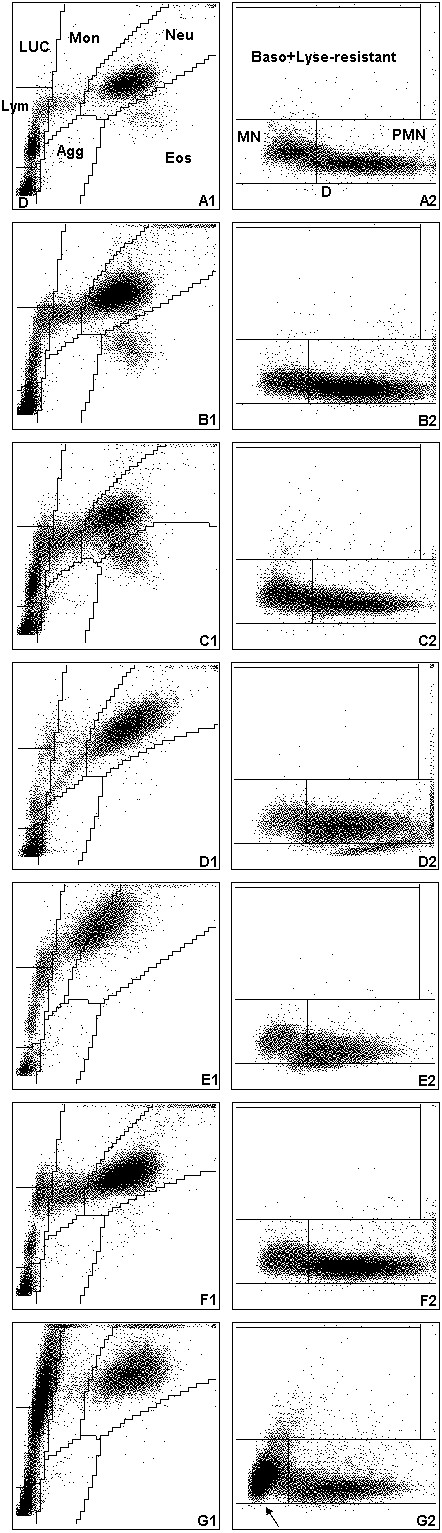
**Cytograms of the ADVIA 120 for dog samples; Lym: lymphocytes; LUC: large unstained cells; Mon: monocytes; Neu: neutrophils; Eos: eosinophils; AGG: platelet aggregates; D: debris; MN: mononuclear cells; PMN: polymorphonuclear cells; Baso: basophils; Lyse-resistant: lyse-resistant cells.** On the left-hand side, the peroxidase scattergrams (myeloperoxidase content on the x-axis and cell size on the y-axis) are depicted; on the right-hand side, baso cytograms (nuclear complexity on the x-axis and cell size on the y-axis) are shown. **(A)** Normal cytograms. **(B)** Suspicion of left shift: in the baso cytogram **(B2)**, the mononuclear and polymorphonuclear cells are not well separated (“worm with swollen neck”). **(C)** No clear separation of cell populations: in the peroxidase cytogram **(C1)**, neutrophils and eosinophils are not clearly separated. **(D)** No clear separation of cell populations and suspicion of reactive lymphocytes: in the peroxidase cytogram **(D1)**, a separate cell population extending from the lymphocyte gate into the LUC gate is seen. **(E)** No clear separation of cell populations and suspicion of left shift: in the peroxidase cytogram **(E1)**, the neutrophil population is shifted towards the upper left of the neutrophil gate, extending into the monocyte gate, which is indicative of myeloperoxidase deficiency of neutrophils. In the baso cytogram **(E2)**, a shortened population of polymorphonuclear cells is present. **(F)** Suspicion of reactive lymphocytes: in the peroxidase cytogram **(F1)**, a separate cell population extending from the lymphocyte gate into the LUC gate indicating atypical lymphocytes or blasts is seen; in the baso cytogram **(F2)**, however, no major abnormalities are detected. **(G)** Suspicion of blasts: in the peroxidase cytogram **(G1)**, a large cell population extending from the lymphocyte gate into the LUC gate indicating blast cells are present; in the baso cytogram **(G2)** a “blast nose” can be identified (arrow) and cells are scattering from the mononuclear area into the lyse-resistant area.

**Figure 2:**
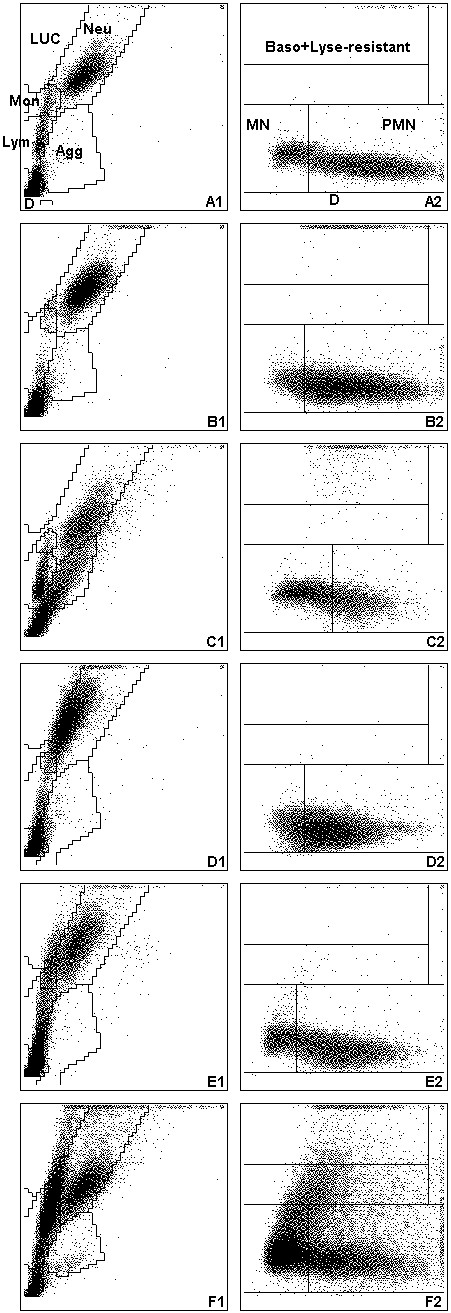
**Cytograms of the ADVIA 120 for cat samples.** See Figure 1 for remainder of key. **(A)** Cytograms without any abnormalities. **(B)** Suspicion of left shift: In the baso cytogram **(B2)**, an indistinct separation between the mononuclear and polymorphonuclear cell populations is noted. In the peroxidase cytogram **(B1)**, few cells are scattered upwards from the neutrophil population, extending the scale of the y-axis and accumulating on the upper boundary of the cytogram, which indicates large and possibly toxic neutrophils. **(C)** No clear separation of cell populations: in the peroxidase cytogram **(C1)**, numerous platelet aggregates are noted spreading into the neutrophil and lymphocyte gate. In the baso cytogram **(C2)**, a “shortened” population of polymorphonuclear cells indicative of a left shift is present. **(D)** No clear separation of cell populations and suspicion of left shift: in the peroxidase cytogram **(D1)**, the neutrophil population is shifted towards the upper left of the neutrophil gate, extending into the LUC gate, which is indicative of myeloperoxidase deficiency of the neutrophils. In the baso cytogram **(D2)**, polymorphonuclear and mononuclear cells are not clearly separated. **(E)** Suspicion of atypical lymphocytes: in the peroxidase cytogram **(E1)**, an increased number of cells are present in the LUC gate. In the baso cytogram **(E2)**, few cells are scattered from the mononuclear area upwards into the lyse-resistant area. **(F)** Suspicion of blasts: in the peroxidase cytogram **(F1)**, a large cell population extending from the lymphocyte gate into the LUC gate indicating blast cells are present; in the baso cytogram **(F2)**, many cells are scattered from the mononuclear area into the lyse-resistant area.

**Figure 3:**
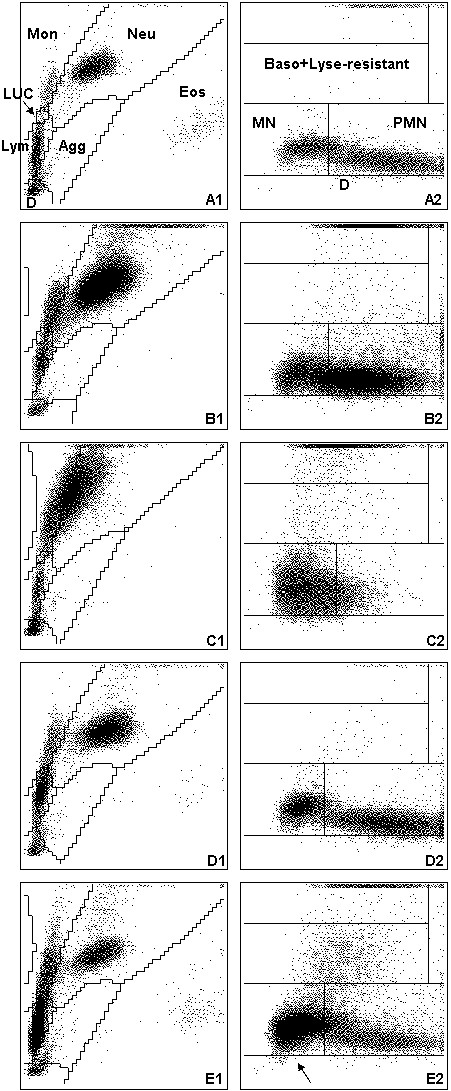
**Cytograms of the ADVIA 120 for horse samples.** For remainder of key see Figure 1. **(A)** Cytograms without any abnormalities. **(B)** Suspicion of left shift: in the baso cytogram **(B2)**, an indistinct separation between the mononuclear and polymorphonuclear cell populations is noted (“worm with swollen neck”). In the peroxidase cytogram **(B1)**, few cells are scattered upwards from the neutrophil population indicating large and possibly toxic neutrophils. **(C)** No clear separation of cell populations and suspicion of left shift and myeloperoxidase deficiency of neutrophils. **(D)** Suspicion of atypical lymphocytes: in the peroxidase cytogram **(D1)**, an increased number of cells are present in the LUC gate. In the baso cytogram **(D2)**, few cells are scattered from the mononuclear area upwards into the lyse-resistant area. **(E)** Suspicion of blasts: in the peroxidase cytogram **(E1)**, a large cell population extending from the lymphocyte gate into the LUC and monocytes gate indicating blast cells are present; in the baso cytogram **(E2)**, a “blast nose” (arrow) can be identified and cells are scattered from the mononuclear area into the lyse-resistant area.

Both clinics primarily see referred cases with acute or chronic illnesses. All CBCs were performed at the day of submission with the ADVIA 120 (Siemens, Healthcare Diagnostics GmbH, formerly Bayer) using the veterinary software version 5.3.1.-MS. The ADVIA 120 is a flow cytometry-based haematology instrument that uses two separate methods to analyse leukocytes [18]. In the peroxidase channel, the red blood cells (RBCs) are lysed and peroxidase reagents are used to distinguish between peroxidase-positive cells (neutrophils, eosinophils (except cat) and monocytes) and peroxidase-negative cells (lymphocytes, rubricytes and basophils). Results are graphically displayed in a peroxidase cytogram (Figures 1, 2, 3 A1). Here, cell size (y-axis) is plotted versus peroxidase activity (x-axis) and species-specific gates are applied automatically (Figures 1, 2, 3 A1). In the baso channel, RBCs and platelets (PLTs) are lysed and the cytoplasmic membranes of all leukocytes except basophils are stripped away. Cells are then classified according to their size and lobularity/nuclear density. By measuring the lobularity and nuclear density, information about the degree of maturity of each leukocyte nucleus is gained [19]. In the baso cytogram, cell size (y-axis) is plotted versus lobularity/nuclear density (Figures 1, 2, 3 A2).

In our laboratory, medical validation of peroxidase and baso cytograms is routinely performed by a board-certified clinical pathologist (NB) or vets performing a training program of the European College of Veterinary Clinical Pathology (ECVCP). Manual differentials were requested based on defined criteria, which are shown in Table 1. These criteria are based on many years of experience with the ADVIA 120 cytograms and with the knowledge of cytogram appearance in healthy animals and animals with specific morphological abnormalities and diseases [1]. ADVIA120 cytograms of all samples in which a manual differential was performed were reviewed retrospectively by one of the authors (MS) to reduce inter-observer variation. Causes for request of a manual differential were verified and classified into four different categories (Table 1). Multiple causes per sample were possible. Sensitivity and specificity of the detection of left shift based on ADVIA 120 cytograms were evaluated for all samples in which a manual differential was requested. More than 0.5x10^9^/l bands based on the 100-cell manual differential were considered a left shift.

**Table 1:** Classification of the causes for requesting manual leukocyte differentials

**Cause for manual differential**	**Characteristic findings in the ADVIA 120 cytograms**
1) Suspicion of left shift	- Indistinct separation of polymorphonuclear and mononuclear cells (“worm with swollen neck”)
	- Shortened population of polymorphonuclear cells (“short worm”) in the ADVIA 120 baso cytogram
2) Inappropriate separation of cell populations	- Indistinct circumscription or missing separation of the cell populations in the ADVIA 120 peroxidase cytogram
	- Evidence of myeloperoxidase-deficient neutrophils
	- Cells spreading from the mononuclear or polymorphonuclear gate into the lyse-resistant cell area (baso cytogram)
	- Discrepancy between the peroxidase and baso cytogram (Figure 3C)
3) Suspicion of atypical lymphocytes	- Increased number of cells in the large unstained cell (LUC) gate
4) Suspicion of abnormal cells (e.g. blasts)	- Increased number of cells in the LUC gate
	- Cells spreading from the mononuclear or polymorphonuclear gate into the lyse-resistant cell area (baso cytogram)
	- Evidence of a “blast nose” in the baso cytogram

Furthermore, the number of false positive alerts – i.e. the number of samples in which a manual differential was requested based on abnormal ADVIA 120 cytograms but neither left shift nor morphological abnormalities in the leukon were observed on the blood smear – was determined for a subgroup of specimens (436 dog, 153 cat and 76 horse) in which a manual differential count was requested and additional information about morphological changes of leukocytes (e.g. toxic neutrophils, reactive lymphocytes) was available.Examples for typical ADVIA 120 cytograms requiring a manual differential are depicted in Figures 1, 2, 3 for dogs, cats and horses.

### Statistical analysis

The percentage of requested manual differentials and underlying causes were determined separately for dogs, cats and horses. To verify statistically significant differences between species, chi-square tests were performed.

## Results

As shown in Table 2, a manual differential was requested in about 20% of the dog and horse samples based on our review criteria (Table 1). In cat samples, this percentage was higher, which was mainly due to a higher proportion of samples with suspected left shift compared to other species (Table 2). This difference, however, was not significant (p = 1.00). Additionally, indistinct separation of cellular populations was present more often in cats (Table 2), but the difference was also not significant (p = 0.56). In all species, the main reason for requesting a manual differential was the suspicion of a left shift (Table 2). In dog samples, reactive lymphocytes were suspected more frequently than in cats and horses (Table 2); however, the difference was not significant (p = 1.00). Atypical blasts were suspected infrequently in all species (p = 1.00; Table 2).

**Table 2:** Proportion of manual differentials requested by clinical pathologists between 2004 and 2006 based on defined criteria in 14,953 dog, cat and horse blood samples

	**Dogs**	**Cats**	**Horses**
Absolute sample number	10,139	2,494	2,320
Manual differentials in %	21	32	20
Reasons for requesting manual differentials (%)			
Suspicion of left shift	13	25	17
No clear separation of cell populations	10	15	10
Suspicion of reactive lymphocytes	12	5	2
Suspicion of blasts	0.3	0.2	0.0009

Sensitivity and specificity of the detection of left shift based on ADVIA 120 cytograms are shown in Table 3. The number of false positive alerts regarding the presence of a left shift or morphological changes of the leukon based on the morphology of the ADVIA 120 cytograms is shown in Table 4.

**Table 3:** Sensitivity and specificity of detecting a left shift based on the morphology of ADVIA 120 cytograms in dog, cat and horse blood samples

	**Dogs**	**Cats**	**Horses**
Sensitivity (%)	80	94	97
Specificity (%)	49	24	18

**Table 4:** Proportion of false positive alerts regarding left shift and abnormalities of the leukon based on the ADVIA 120 cytogram of dog, cat and horse blood samples with available manual differential and evaluation of the leukocyte morphology on the blood smear

	**Dogs**	**Cats**	**Horses**
Absolute sample number	436	153	76
False positive alerts (absolute number)	57	46	23
False positive alerts (%)	13	30	30

## Discussion

The results of our study are comparable to human studies reporting a rate of manual slide review of approximately 25% [11,12]. However, in the previous studies, the slide review included both a single microscopic examination without manual leukocyte differentiation as well as blood smear review and manual leukocyte differentiation. Furthermore, blood smear reviews were not only requested due to abnormalities in the leukon, but also due to abnormalities in the erythron or thrombon [11] as well as the occurrence of instrument flags indicating the presence of blasts, immature cells or atypical cells [12]. In our laboratory, requesting a blood smear review was always due to suspected abnormalities in the leukon and always accompanied by manual leukocyte differentiation, while abnormalities in the erythron and thrombon were assessed by clinical pathologists without performing a manual leukocyte differential. However, in contrast to the human studies, cytograms rather than instrument flags were used to detect the potential presence of blasts, immature or atypical cells as the flag algorithms are not well established in veterinary medicine [1].

It is notable that, in our study, a manual differential was requested in 32% of feline samples but only in about 20% of canine and equine samples. The most likely reason is the higher prevalence of platelet aggregates in feline samples, which have been reported in 64% of feline samples [20]. Platelet aggregates interfere with the separation of cellular populations in the peroxidase scattergram (Figure 2 C1). In such samples, platelet aggregates may also interfere with the separation of mononuclear and polymorphonuclear cells in the baso cytogram and thus mimicking a left shift (Figure 2 C2). The presence of platelet aggregates is also the most likely reason for the low specificity in feline samples (24%) of correctly recognising samples with left shift based on the ADVIA cytograms. The specificity of equine samples was comparably low (18%). A previous study evaluating a predecessor of the ADVIA 120, the Technicon H1-E analyser, reported that this kind of pattern in the baso cytogram (“short worm”) occurred in samples with prominent toxic neutrophils even without a left shift [15]. As the technology of the Technicon H1-E analyser and the ADVIA 120 for the WBC differentiation is comparable, this underlying cause has to be considered. However, in our experience, there are also samples showing the described pattern in the baso cytogram in the absence of any morphological changes of leukocytes. A possible reason may be a different susceptibility of horse leukocytes to the reagent used in the baso channel or the presence of immature neutrophils with only slightly more segmentation than band neutrophils – however, further studies are needed to verify possible underlying causes for this phenomenon.

In dog samples, the specificity of the ADVIA 120 cytograms to detect bands was higher than for cat and horse specimens, but still low with a value of 49%. However, during medical validation of ADVIA cytograms, high sensitivity is considered more important than high specificity as false positives are undesirable, but false negatives are potentially fatal for the patient. Sensitivity ranging between 80% and 97% for different species appears satisfactory for clinical purposes, so the ADVIA 120 cytograms can be considered to be a good screening tool for detection of left shift. Besides the presence of left shifts, the detection of morphological changes is an important clinical requirement. Based on our data, however, we cannot offer any information about the sensitivity of ADVIA 120 cytograms to predict the presence of morphologically abnormal leukocytes on the blood smear. However, typical abnormalities have been described for animals and correlated with specific clinical conditions [1,14,15]. Furthermore, the number of false positive alerts – e.g. specimens with requested manual differential, but an absence of morphological changes or left shift – is fairly low in dog samples (13%). In cat and horse samples, the values are higher (30%). The main cause is most likely the suspicion of left shifts based on cytogram assessment (see above).

Lantis et al. showed that laboratory operations could be optimised by modifying the algorithm for performing leukocyte differentials [13]. Instead of establishing criteria for manual differentiation, the authors proposed criteria for blood smear review. Based on the results of blood smear review, manual differentials are performed or the automated differential is reported [13]. In veterinary medicine, experts’ opinions about blood smear reviews are diverse. Whereas in some laboratories, manual differentials are performed based on the findings of blood smear scans, others perform and report manual differentials in every sample, even if the automated differential is accurate. In human clinical pathology laboratories, however, the latter procedure was reported to result in an increased laboratory workload, and a reduction in laboratory productivity and precision of the white blood cell differential [11,21]. In our laboratory, the criteria for performing manual white blood cell differentials were based on the interpretation of ADVIA 120 cytograms [1] during medical validation. With this approach, about a quarter of complete blood cell counts require manual differentiation. If we additionally applied the approach proposed by Lantis et al. [13] and perform manual differentials only on the slides where the automated differential is obviously inaccurate or atypical cells are present, the number of manual differentials could be further reduced. A reduction of the number of manual differentials would be especially possible in cat and horse samples as the number of specimens with suspected left shift according to the cytogram evaluations but absence of bands on the blood smear was relatively high. In these specimens, blood smear reviews would have been sufficient to rule out left shift and avoid manual differentiation. The inability of the ADVIA 120 to detect rubricytes could be an issue. In 6% (134 specimens) of the dog and 2% (19 specimens) of the cat samples with a manual differential of ≥10 rubricytes/100 WBC. The cytograms of these samples revealed no consistent abnormalities. Thus, specimens with inappropriate rubricytosis could possibly be missed with our approach. However, inappropriate rubricytosis was only present in four of the 134 dog samples with ≥10 rubricytes/100 WBC. In the remaining 130 samples, the patients were recovering from anaemia or were still anaemic.

Moreover, it has to be kept in mind that automated haematology analysers such as the ADVIA 120 are unable to reliably detect blood parasites and that the number of false negative alerts (normal ADVIA 120 cytogram but abnormalities on the blood smear) has not yet been investigated. Thus, a microscopic evaluation of the blood smear remains mandatory in all clinically ill patients.

The specimens evaluated in this study originate from university clinics with primarily referred cases. Different results can be expected from laboratories receiving a higher percentage of specimens taken for health checks or screening examinations. Furthermore, the approach for validating automated differentials depends very much on the analyser, the experience of clinical pathologists available in the laboratory and the sample age. Concerning the leukon, previous studies evaluating the effects of sample storage reported an increase in the proportion of mononuclear cells using the basophil method [22,23]. The number of cells displayed in the lyse-resistant area also increased after 24 and 48 hours [22]. Thus, assessment of cytograms of stored samples to define the necessity of manual differentials or blood film scans may be difficult and more studies are needed with the focus on cytogram changes caused by storage of samples.

The study is limited by its retrospective nature and the fact that a manual differential count and microscopic evaluation of abnormalities of the leukon were not performed for samples with normal ADVIA 120 cytograms. Besides assessment of the cytogram, evaluation of flagging options would be interesting for future investigations.

Thus, further prospective studies are needed to verify the sensitivity of cytograms and flagging options of different advanced haematology analysers, not only concerning the accuracy of the differential count, but also regarding the detection of morphological changes of the white blood cells. With this knowledge, the strengths of the advanced haematology analysers could be used to further reduce the workload in clinical pathology laboratories.

## Conclusions

The rate of manual slide reviews observed in our study was comparable to the results published for human laboratories. Based on our results it can be concluded that – similar to human laboratories – faster and more cost-effective procedures would be possible in veterinary laboratories if microscopic examination was used as a validation procedure rather than as a reflexive substitute for automated differentiation.

## Abbreviations

AGG: Platelet aggregates; Baso: Basophils; D: Debris; CBC: Complete blood cell count; Eos: Eosinophils; Lym: Lymphocytes; LUC: Large unstained cells; Lyse-resistant: Lyse-resistant cells; MN: Mononuclear cells; Mon: Monocytes; Neu: Neutrophils; PMN: Polymorphonuclear cells; PLT: Platelets; RBC: Red blood cells; WBC: White blood cells.

## Competing interests

The authors declare no competing interests.

## Authors’ contributions

MS, AM and NB designed the study and analysed the data. MS and NB performed the experiments and wrote the paper. All authors read and approved the final manuscript.
